# Financial Hardship and Nonadherence to Lifestyle and Surveillance in Childhood Cancer Survivors

**DOI:** 10.1001/jamanetworkopen.2026.15527

**Published:** 2026-05-29

**Authors:** Neel S. Bhatt, Fang Wang, Shizue Izumi, Yan Chen, Timothy J. D. Ohlsen, Gregory T. Armstrong, I-Chan Huang, Anne Kirchhoff, Elyse R. Park, Claire Snyder, K. Robin Yabroff, Yutaka Yasui, Paul C. Nathan

**Affiliations:** 1 Clinical Research Division, Fred Hutchinson Cancer Center, Seattle, Washington; 2 Department of Pediatrics, University of Washington, Seattle; 3 St Jude Children’s Research Hospital, Memphis, Tennessee; 4 Faculty of Data Science, Shiga University, Hikone, Japan; 5 Huntsman Cancer Institute and Department of Pediatrics, University of Utah, Salt Lake City; 6 Department of Psychiatry, Massachusetts General Hospital, Boston; 7 Department of Medicine, Johns Hopkins University School of Medicine, Baltimore, Maryland; 8 American Cancer Society, Atlanta, Georgia; 9 The Hospital for Sick Children, The University of Toronto, Toronto, Ontario, Canada

## Abstract

**Importance:**

The association between medical financial hardship and nonadherence to healthy lifestyle and late-effects surveillance recommendations in long-term survivors of childhood cancer is unknown.

**Objective:**

To study the associations between medical financial hardship and nonadherence to healthy lifestyle and late-effects surveillance recommendations.

**Design, Setting, and Participants:**

This retrospective cohort study was performed among participants in the multiinstitutional Childhood Cancer Survivor Study. Participants were 5-year cancer survivors diagnosed at age 21 years or younger between January 1, 1970, and December 31, 1999, and who completed both a medical financial hardship survey between 2017 and 2019 and a follow-up survey assessing lifestyle behaviors and adherence to risk-based surveillance between 2020 and 2022. Data were analyzed between September 29, 2023, and September 2, 2025.

**Exposure:**

Self-reported medical financial hardship determined by an affirmative response to at least 1 item in the material, behavioral, or psychological domains.

**Main Outcomes and Measures:**

Associations of non–guideline-concordant physical activity, problematic drinking, smoking, and abnormal body mass index with domains of medical hardship were examined using separate multivariable logistic regression models. Associations between a composite lifestyle score (unhealthy, moderately healthy, and healthy) and nonadherence to surveillance for cardiomyopathy or breast, colorectal, cervical, and/or skin cancer were examined using polytomous logistic regression models.

**Results:**

Among 3322 survivors who completed both surveys (median [range] age, 41 [20-69] years; 1751 female [52.7%]), the presence of material, behavioral, and psychological hardship was reported by 1401 (42.2%), 1003 (30.2%), and 1243 (37.4%), respectively. Material hardship was associated with a greater odds of non–guideline-concordant physical activity (odds ratio [OR], 1.67 [95% CI, 1.29-2.18]) and abnormal body mass index (OR, 1.47 [95% CI, 1.15-1.88]). Behavioral and psychological hardships were associated with a higher odds of smoking (OR, 2.29 [95% CI, 1.13-4.62] and 3.95 [95% CI, 2.42-6.44], respectively). Material and psychological hardship were associated with a composite unhealthy lifestyle score (OR, 1.52 [95% CI, 1.11-2.07] and 1.96 [95% CI, 1.31-2.93], respectively), and a higher risk was noted among survivors reporting hardship in at least 2 domains. Psychological hardship was associated with greater nonadherence to skin cancer surveillance (OR, 1.78 [95% CI, 1.05-3.02]) among survivors at high risk due to treatment exposures. Material hardship was associated with greater nonadherence to breast cancer screening (OR, 2.85 [95% CI, 1.27-6.38]) among survivors at average risk. Associations between multiple medical hardship domains and nonadherence to cervical cancer screening were also observed (material and behavioral hardship: OR, 3.20 [95% CI, 1.44-7.14]; material and psychological hardship: OR, 2.18 [95% CI, 1.10-4.35]; behavioral and psychological hardship: OR, 3.25 [95% CI, 1.50-7.04]).

**Conclusions and Relevance:**

This cohort study of adult survivors of childhood cancer found that medical financial hardship was associated with nonadherence to healthy lifestyle behaviors and certain recommended surveillance tests for subsequent malignant neoplasms. These findings underscore the need to identify and address medical financial hardship as a potential risk factor of nonadherence to healthy lifestyle and guideline-concordant survivorship care.

## Key Points

**Question:**

Is there an association between medical financial hardship and adherence to healthy behavior and recommended surveillance among adult survivors of childhood cancer?

**Findings:**

In this cohort study of 3322 adults previously treated for a childhood cancer who were more than 3 decades from their diagnosis, more than one-half reported medical financial hardship. Medical financial hardship was associated with nonadherence to healthy lifestyle behaviors and certain recommended surveillance tests for subsequent malignant neoplasms.

**Meaning:**

These findings underscore the need to identify and address medical financial hardship as a potential risk factor of nonadherence to a healthy lifestyle and guideline-concordant survivorship care.

## Teaser

This cohort study examines the association between medical financial hardship and nonadherence to healthy lifestyle behaviors and surveillance recommendations among adult survivors of childhood cancer.

## Introduction

With advances in childhood cancer therapies and supportive care, 5-year survival rates now exceed 85%,^1^ yielding a population of more than half a million survivors of childhood cancer in the US.^2^ Survivors’ treatment exposures elevate not only their risk of mortality but also their likelihood of developing physical and psychological morbidities that hinder educational attainment, employment, income stability, health insurance coverage, and access to health care.^3,4^

Studies of survivors of adult cancers have shown that compared with individuals without a cancer history, survivors are more likely to experience medical financial hardship, a broad term encompassing difficulties related to the cost of medical care.^5^ This increased risk has been reported in material (problems with paying for medical expenses or bills), psychological (worry about medical costs), and behavioral (delaying medical care because of cost) domains.^6,7,8,9,10,11,12^ Similarly, studies of medical financial hardship among adult survivors of childhood cancers have reported significantly greater hardship among survivors compared with siblings or the general population,^13,14,15,16^ with the risk further elevated among those with lower educational attainment or without private health insurance.^16^

Medical financial hardship has been associated with social drivers of health, including food and housing insecurity, utility instability, and transportation barriers, which could constrain survivors’ ability to maintain a healthy lifestyle, including a healthy diet and routine health care.^11^ Indeed, survivors of adult cancers with high medical financial hardship have reported low use of preventive or screening services, including influenza vaccination and breast cancer screening.^12^ While it is known that a substantial proportion of adult survivors of childhood cancer do not receive survivor-focused care,^17^ undergo recommended surveillance for subsequent malignant neoplasms^18^ and late cardiovascular sequelae,^19^ or participate in routine physical exercise,^20^ the current literature lacks information on the associations between medical financial hardship and health behaviors and receipt of preventive or surveillance services in this population.

To address this knowledge gap, we identified individuals from the Childhood Cancer Survivor Study (CCSS) to examine the association between medical financial hardship and survivors’ adherence to a healthy lifestyle and risk-based surveillance recommendations. We hypothesized that survivors who reported medical financial hardship would be more likely to report unhealthy lifestyle (physical inactivity, smoking, problematic drinking), abnormal body mass index (BMI [calculated as weight in kilograms divided by height in meters squared]), and nonadherence to surveillance for subsequent malignant neoplasms and cardiomyopathy compared with those not reporting medical financial hardship.

## Methods

### Study Design and Participants

This cohort study used data from the CCSS, a National Cancer Institute–funded, multiinstitutional, retrospectively constructed cohort study with prospective follow-up of childhood cancer survivors. The protocols and questionnaires used by the CCSS underwent approval by the Institutional Review Boards of all collaborating centers, and participants provided written informed consent. Our study was reviewed and approved by the CCSS Research Publications Committee. This study followed the Strengthening the Reporting of Observational Studies in Epidemiology (STROBE) reporting guideline for cohort studies.

Eligible survivors were diagnosed at age 21 years or younger between January 1, 1970, and December 31, 1999, at 1 of 31 institutions in the US and Canada and had survived at least 5 years after diagnosis. Detailed descriptions of the CCSS study design, procedures, and follow-up surveys have been published previously.^21,22^ Treatment details were abstracted from institutional medical records, and survivors provided self-reported data on sociodemographics (age, sex, race and ethnicity), health behaviors, chronic health conditions, and quality of life through periodic follow-up surveys. Race and ethnicity were assessed in this study because of potential differences in medical financial hardship by race and ethnicity. Participants were grouped into non-Hispanic White and non-White (American Indian or Alaska Native, Asian or Pacific Islander, Black, and multiracial) race and ethnicity because of the limited number of participants in categories other than non-Hispanic White. For this analysis, we focused on a subgroup of the CCSS cohort who were selected through age-stratified and cancer diagnosis–stratified sampling for a medical financial hardship survey between 2017 and 2019.^16^ Participants who responded to the medical financial survey also were asked to respond to a survey administered between 2020 and 2022 that assessed lifestyle behaviors and adherence to recommended surveillance. This group formed the study sample for the current analysis. Sample size was determined by the number of eligible CCSS participants completing both surveys; no formal power calculation was performed.

### Survey Domains and Outcomes

eTable1 in Supplement 1 details the survey domains, covariates, and outcomes. These surveys are publicly available on the CCSS website.^23^ Survivors’ responses to 19 medical financial hardship survey items were grouped into 3 medical financial hardship domains: material (8 items, eg, high out-of-pocket costs), behavioral (8 items, eg, delaying care due to cost), or psychological (3 items, eg, worry about financial situation) using principal component analysis, as described previously.^16^ Presence of medical financial hardship was determined by an affirmative response to at least 1 item in any of the domains. Chronic health conditions were self-reported, with age at the first onset ascertained from the CCSS surveys, and graded per the National Cancer Institute’s Common Terminology Criteria for Adverse Events, version 4.03 as follows: mild (grade 1), moderate (grade 2), severe or disabling (grade 3), life threatening (grade 4), or fatal (grade 5).^24,25^ A chronic health condition burden score was derived according to the number and grade of chronic health conditions^26^ and categorized as no or low (0), medium (1), high (2), and very high (3).

Self-reported physical activity scores were calculated as the frequency of moderate-intensity or vigorous-intensity exercise sessions per week within the past month multiplied by the average reported duration in minutes, weighted by corresponding metabolic equivalent task (MET), and expressed as average MET-hours per week. As published previously, estimates of 5 MET-hours/week and 9 MET-hours/week were used for moderate and vigorous activity, respectively.^27^ The outcome was dichotomized based on the following current national physical activity recommendations for adult cancer survivors: less than 9 MET-hours/week (not meeting guidelines for vigorous exercise per week) vs 9 MET-hours/week or more (meeting guidelines). A MET-hour per week value of 9 is approximately 150 minutes of moderate to vigorous exercise per week, which is the current national recommendations per Centers for Disease Control and Prevention. Self-reported problematic drinking was measured according to the following National Institute on Alcohol Abuse and Alcoholism definitions: for men, consuming 5 drinks or more on any day or 15 drinks or more per week within the past 12 months; for women, consuming 4 drinks or more on any day or 8 drinks or more per week within the past 12 months.^28^ Smoking status was determined by self-report of lifetime smoking of 100 cigarettes or more and current smoking behavior, and dichotomized as current vs former or never smoker. Body mass index was derived from self-reported height and weight; categorized as underweight (<18.5), normal weight (18.5-24.9), overweight (25.0-29.9), and obese (≥30 kg/m^2^); and dichotomized as abnormal (underweight, overweight, obese) vs normal.

Using survivors’ responses for physical activity, drinking, smoking, and BMI, a composite lifestyle score was calculated.^29^ To derive the score, physical activity was scored as 0 (0 MET-hours/week), 0.5 (3-6 MET-hours/week), or 1 (>6 MET-hours/week); smoking as 0 (current or former smoker) or 1 (never smoker); drinking as 0 (problematic) or 1 (nonproblematic), and BMI as 0 (abnormal) or 1 (normal). Scores of 0 to 2.0, 2.5 to 3.0, and 3.5 to 4.0 represented unhealthy, moderately healthy, and healthy lifestyles, respectively.

Self-reported adherence to recommended surveillance for subsequent malignant neoplasms and cardiomyopathy was measured for survivors at high risk for breast cancer, colorectal cancer, skin cancer, and cardiomyopathy based on exposure to radiation and/or anthracyclines in accordance with the Children’s Oncology Group (COG) Long-Term Follow-Up Guidelines, version 5.0, which were published in October 2018 (eTable 2 in Supplement 1).^30^ Cumulative anthracycline dose was derived using a previously published doxorubicin equivalent dose calculation method (eTable 2 in Supplement 1).^31^ Survivors were classified as adherent if they completed the recommended surveillance within the time frame recommended by the COG Long-Term Follow-Up guidelines. Adherence to general population cancer screening was also measured among the remaining survivors (average risk) by comparison with the American Cancer Society screening recommendations (2020-2022) for colorectal, breast, and cervical cancer (eTable 2 in Supplement 1), consistent with prior research.^18^

### Statistical Analysis

Survivors’ sociodemographic and clinical characteristics are described using frequencies and percentages for categorical variables and medians and ranges for continuous variables. Separate multivariable logistic regression models were used to examine associations between domains of medical financial hardship and not meeting physical activity guidelines and reporting problematic drinking, smoking, and abnormal BMI. A polytomous logistic regression model was used to examine associations between domains of medical financial hardship and a composite lifestyle score (unhealthy, moderately healthy, or healthy). Models were also constructed to study the associations between domains of medical financial hardship and nonadherence to breast, colorectal, or skin cancer or cardiomyopathy surveillance per the COG Long-Term Follow-Up guidelines among high-risk survivors, as well as breast cancer, colorectal, or cervical cancer screening per American Cancer Society screening recommendations for the rest of the survivors. Each model was adjusted for age at the time of the most recent survey, sex, race and ethnicity, highest education level, and chronic health condition burden. For each analysis, the effects of individual medical financial hardship domains, hardship in 2 domains (material and behavioral, material and psychological, or behavioral and psychological), and hardship in all 3 domains were examined. Interactions between medical financial hardship and other covariates were not studied. For the analysis focused on adherence to surveillance and screening recommendations, survivors who had developed 1 of the target cancers as a subsequent malignant neoplasm (skin, colorectal, breast, or cervical) were excluded from the analysis. Similarly, survivors who developed grade 3 or 4 congestive heart failure were excluded from analysis of cardiomyopathy surveillance adherence. For each model, a complete case analysis approach was used. The percentage of participants with missing data was less than 10% for all the models. All tests were 2-sided, and *P* < .05 was considered statistically significant. Analyses were performed using SAS, version 9.4 (SAS Institute Inc).

## Results

### Characteristics of Study Participants

Of 5488 CCSS participants approached to complete the medical financial hardship survey between 2017 and 2019, 3555 (64.7%) responded, and of those, 3322 (93.4%) responded to the survey administered between 2020 and 2022 and were included in our analysis (median [range] age, 41 [20-69] years; 1751 female [52.7%] and 1571 male [47.2%]; 2811 of 3247 identifying as non-Hispanic White [86.5%] and 436 as not White [13.4%]). Table 1 describes the sociodemographic and treatment characteristics of survivors. Survivors were a median of 34.4 years (range, 29.7-51.4 years) since diagnosis, and 1886 (56.7%) were aged 40 years or older at the time of their survey assessing lifestyle behaviors and adherence to recommended surveillance. The most common diagnoses were leukemia (1028 participants [30.9%]) and central nervous system tumors (528 participants [15.8%]). Most survivors had a college degree or higher (2092 of 3313 [63.1%]) and were covered by either private (2474 of 3318 [74.5%]) or public (599 of 3318 [18.0%]) health insurance. A total of 778 participants (23.3%) had a high or very high chronic health condition burden at the time of the 2020-2022 survey.

**Table 1.** Sociodemographic and Clinical Characteristics of Survivors of Childhood Cancer (N = 3322)^a^

Characteristic	All survivors, No. (%)	Survivors with hardship, No. (%)^b^		
		Material (n = 1401)	Behavioral (n = 1003)	Psychological (n = 1243)
Age at diagnosis, y				
0-4	1283 (38.6)	523 (37.3)	384 (38.3)	492 (39.6)
5-9	731 (22.0)	325 (23.2)	231 (23.0)	295 (23.7)
10-14	764 (23.0)	328 (23.4)	228 (22.7)	274 (22.0)
≥15	544 (16.3)	225 (16.1)	160 (15.9)	182 (14.6)
Age at the time of the 2020-2022 survey completion, y				
18-29	333 (10.0)	145 (10.3)	100 (10.0)	142 (11.4)
30-39	1103 (33.2)	472 (33.7)	360 (35.9)	431 (34.7)
≥40	1886 (56.7)	784 (56.0)	543 (54.1)	670 (53.9)
Time since diagnosis, median (range), y	34.4 (19.7-51.4)	34.1 (19.8-51.4)	33.5 (19.9-51.4)	33.8 (19.8-51.4)
Sex				
Female	1751 (52.7)	786 (56.1)	589 (58.7)	731 (58.8)
Male	1571 (47.2)	615 (43.9)	414 (41.3)	512 (41.2)
Race and ethnicity				
Non-Hispanic White	2811 (86.5)	1168 (85.3)	818 (83.0)	1007 (82.5)
Not White^c^	436 (13.4)	201 (14.7)	168 (17.0)	214 (17.5)
Missing	75	32	17	22
Diagnosis				
Leukemia	1028 (30.9)	472 (33.7)	329 (32.8)	423 (34.0)
Central nervous system tumor	528 (15.8)	230 (16.4)	152 (15.1)	194 (15.6)
Hodgkin lymphoma	393 (11.8)	160 (11.4)	123 (12.3)	130 (10.5)
Non-Hodgkin lymphoma	280 (8.4)	119 (8.5)	87 (8.7)	103 (8.3)
Wilms tumor	328 (9.8)	118 (8.4)	92 (9.2)	111 (8.9)
Neuroblastoma	237 (7.1)	87 (6.2)	65 (6.5)	88 (7.1)
Soft tissue sarcoma	236 (7.1)	86 (6.1)	71 (7.1)	82 (6.6)
Bone cancer	292 (8.7)	129 (9.2)	84 (8.4)	112 (9.0)
Treatment combinations				
No surgery, chemotherapy, or radiation therapy	7 (0.2)	2 (0.1)	2 (0.2)	3 (0.3)
Surgery only	252 (8.0)	98 (7.5)	63 (6.7)	95 (8.1)
Chemotherapy only	343 (10.9)	153 (11.7)	104 (11.1)	127 (10.9)
Radiation therapy only	8 (0.2)	2 (0.1)	4 (0.4)	1 (0.1)
Surgery and chemotherapy	807 (25.8)	307 (23.4)	231 (24.6)	300 (25.7)
Surgery and radiation therapy	258 (8.2)	106 (8.1)	79 (8.4)	93 (8.0)
Chemotherapy and radiation therapy	324 (10.3)	168 (12.8)	121 (12.9)	159 (13.6)
Surgery, chemotherapy, and radiation therapy	1125 (36.0)	475 (36.2)	334 (35.6)	387 (33.2)
Education				
High school or less	574 (17.3)	275 (19.7)	212 (21.2)	250 (20.1)
Some college	647 (19.5)	346 (24.8)	276 (27.5)	326 (26.3)
College degree or higher	2092 (63.1)	776 (55.5)	514 (51.3)	665 (53.6)
Missing	9	4	1	2
Employment				
Full time	2352 (72.2)	927 (67.7)	602 (62.1)	804 (66.4)
Part time	328 (10.0)	118 (8.6)	96 (9.9)	118 (9.7)
Unemployed	577 (17.7)	324 (23.7)	271 (28.0)	288 (23.8)
Missing	65	32	34	33
Annual household income, $				
<40 000	576 (20.5)	351 (29.4)	322 (38.4)	382 (36.4)
≥40 000 to <80 000	713 (25.4)	360 (30.2)	256 (30.5)	337 (32.1)
≥80 000	1512 (53.9)	482 (40.4)	261 (31.1)	330 (31.5)
Missing	521	208	164	194
Insurance				
Uninsured	247 (7.4)	124 (8.9)	139 (13.9)	146 (11.8)
Private	2474 (74.5)	1001 (71.5)	608 (60.7)	813 (65.5)
Public	599 (18.0)	274 (19.6)	255 (25.4)	282 (22.7)
Missing	4	2	1	2
Current living arrangement				
Independent	2758 (83.5)	1137 (81.4)	816 (81.8)	1031 (83.5)
Dependent	544 (16.4)	259 (18.5)	182 (18.2)	204 (16.5)
Missing	20	5	5	8
Chronic health condition burden				
None or low	1015 (30.5)	375 (26.8)	294 (29.3)	353 (28.4)
Medium	1529 (46.0)	653 (46.6)	460 (45.9)	588 (47.3)
High	566 (17.0)	265 (18.9)	173 (17.3)	218 (17.5)
Very high	212 (6.3)	108 (7.7)	76 (7.6)	84 (6.8)

^a^ Childhood Cancer Survivor Study participants who responded to the medical financial hardship survey (2017-2019) and a survey that assessed lifestyle behaviors and adherence to recommended surveillance (2020-2022).

^b^ Number of patients reporting medical financial hardship in each domain regardless of how they reported other hardship. Note that 1405 survivors (42.3%) reported no medical financial hardship.

^c^ Included American Indican or Alaska Native, Asian or Pacific Islander, Black, and multiracial.

### Prevalence of Medical Financial Hardship, Unhealthy Lifestyle Behaviors, and Abnormal BMI

**Figure 1.**
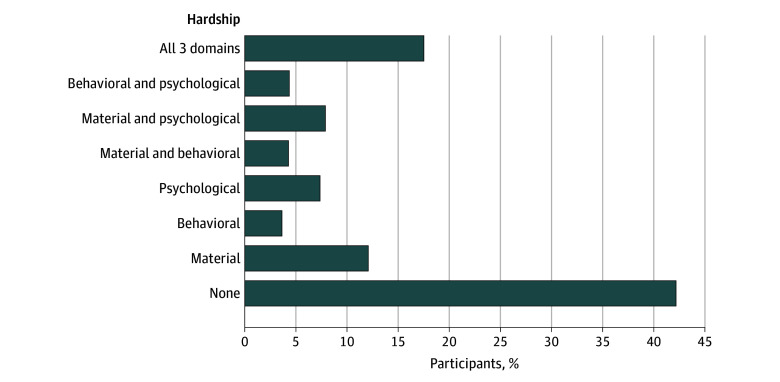
Bar Graph of Medical Financial Hardship Among Childhood Cancer Survivors (N = 3322)

Overall, 1927 survivors (58.0%) reported some form of medical financial hardship (1401 [42.2%] material, 1003 [30.2%] behavioral, 1243 [37.4%] psychological), and 587 (17.6%) reported having hardship in all 3 domains, with 556 (16.7%) having hardship in any 2 domains (material and behavioral, 146 [4.4%]; material and psychological, 263 [7.9%]; behavioral and psychological, 147 [4.4%]) and 774 (23.3%) in only 1 domain (material, 405 [12.2%]; behavioral, 123 [3.7%]; psychological, 246 [7.4%]) (Figure 1). Table 2 highlights self-reported lifestyle behaviors. A total of 2177 of 3205 participants (67.9%) did not meet the recommended guidelines of 9 MET-hours/week. Problematic drinking and smoking behaviors were reported by 32.3% (994 of 3169) and 7.9% (244 of 3072) of survivors, respectively. Abnormal BMI was noted in 65.2% (2114 of 3240) of survivors. Only 22.8% (692 of 3128) of survivors had a healthy lifestyle score, with 37.4% (1133 survivors) and 39.7% (1203 survivors) in the moderately healthy and unhealthy categories, respectively.

**Table 2.** Self-Reported Lifestyle Behaviors of Survivors of Childhood Cancer (N = 3322)

Variable	All survivors, No. (%)	Survivors with hardship, No. (%)^a^		
		Material (n = 1401)	Behavioral (n = 1003)	Psychological (n = 1243)
Moderate or vigorous exercise frequency, median (range), MET-h/wk	5 (0-21)	4 (0-21)	4 (0-21)	4 (0-21)
Met physical activity guidelines				
No (<9 MET-h/wk)	2177 (67.9)	974 (72.4)	673 (70.1)	846 (70.9)
Yes (≥9 MET-h/wk)	1028 (32.0)	371 (27.6)	287 (29.9)	348 (29.2)
Missing	117	56	43	49
Problematic drinking behavior				
Yes	994 (32.3)	392 (29.5)	292 (30.7)	368 (31.5)
No	2075 (67.6)	935 (70.5)	659 (69.3)	802 (68.6)
Missing	153	74	52	73
Smoking status				
Never	2194 (71.4)	862 (67.1)	569 (62.2)	724 (63.6)
Former	634 (20.6)	293 (22.8)	224 (24.5)	258 (22.7)
Current	244 (7.9)	129 (10.0)	122 (13.3)	157 (13.8)
Missing	250	117	88	104
Body mass index^b^				
Underweight (<18.5)	69 (2.1)	30 (2.2)	23 (2.4)	28 (2.3)
Normal (18.5-24.9)	1126 (34.7)	395 (29.0)	276 (28.4)	345 (28.7)
Overweight (25.0-29.9)	1077 (33.2)	418 (30.6)	307 (31.6)	388 (32.2)
Obese (≥30)	968 (29.8)	521 (38.2)	365 (37.6)	443 (36.8)
Missing	82	37	32	39
Lifestyle score				
Unhealthy (0-2.0)	1203 (39.7)	596 (46.9)	445 (49.3)	550 (49.2)
Moderately healthy (2.5-3.0)	1133 (37.4)	450 (35.4)	313 (34.7)	394 (35.2)
Healthy (3.5-4.0)	692 (22.8)	226 (17.8)	144 (16.0)	174 (15.6)
Missing	294	129	101	125

Abbreviation: MET, metabolic equivalent task.

^a^ Number of patients reporting medical financial hardship in each domain regardless of how they reported other hardship. Note that 1405 survivors (42.3%) reported no medical financial hardship.

^b^ Calculated as weight in kilograms divided by height in meters squared.

### Associations Between Medical Financial Hardship and Lifestyle Behaviors and Abnormal BMI

**Figure 2.**
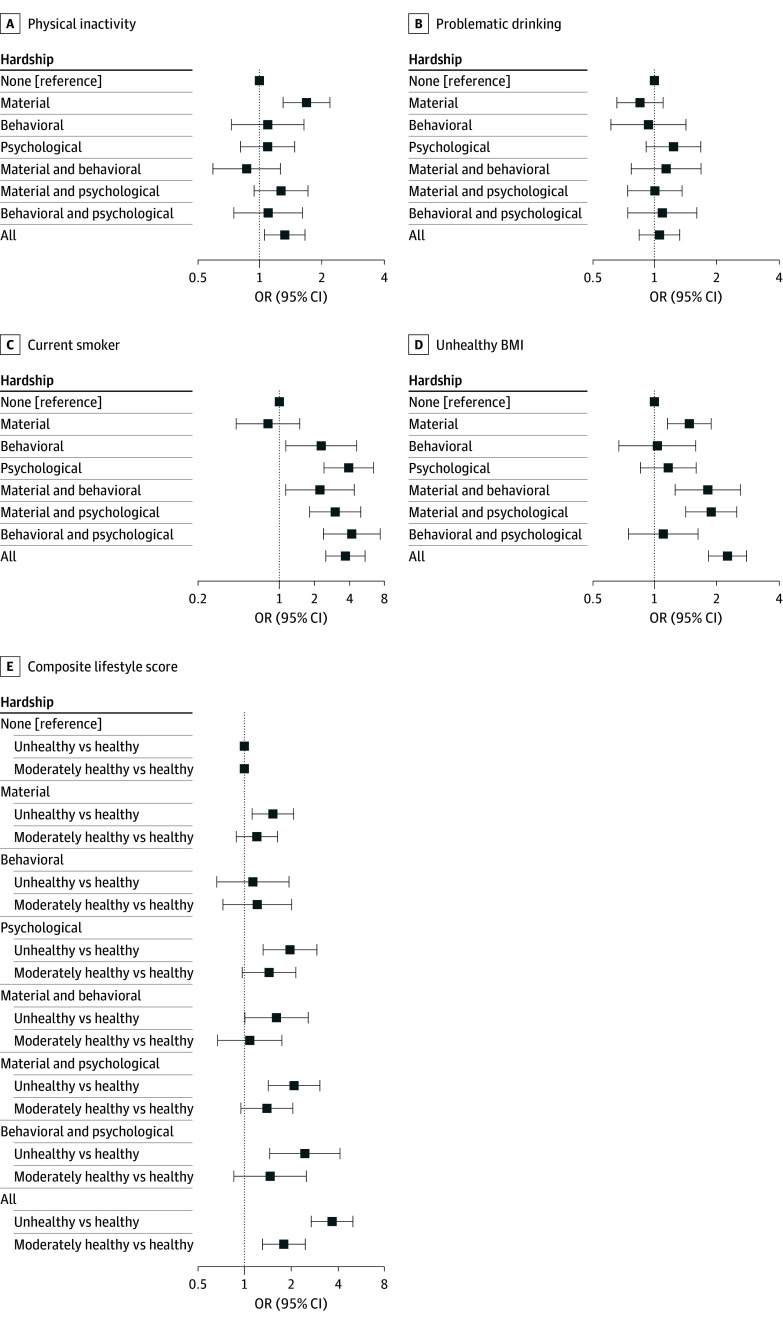
Forest Plots of the Association Between Medical Financial Hardship and Health Behaviors in Childhood Cancer Survivors Odds ratios (ORs) and 95% CIs are provided in eTable 3 in Supplement 1. BMI indicates body mass index.

Figure 2 and eTable 3 in Supplement 1 illustrate the association between medical financial hardship and lifestyle behaviors among study participants. Compared with survivors with no hardship, those reporting material hardship (odds ratio [OR], 1.67 [95% CI, 1.29-2.18]) or all 3 types of hardship (OR, 1.31 [95% CI, 1.04-1.65]) were more likely not to meet physical activity guidelines. Compared with survivors reporting no hardship, those reporting behavioral or psychological hardship or hardship in multiple domains were more likely to report smoking at the time of their 2020-2022 survey (behavioral: OR, 2.29 [95% CI, 1.13-4.62]; psychological: OR, 3.95 [95% CI, 2.42-6.44]; material and behavioral: OR, 2.23 [95% CI, 1.13-4.40]; material and psychological: OR, 3.02 [95% CI, 1.82-5.02]; behavioral and psychological: OR, 4.19 [95% CI, 2.39-7.36]; all 3 types of hardship: OR, 3.70 [95% CI, 2.50-5.46]). Survivors with material hardship also had a greater odds of having an abnormal BMI (OR, 1.47 [95% CI, 1.15-1.88]). A significant association was found for survivors with an unhealthy BMI who had hardship in at least 1 other domain (material and behavioral: OR, 1.81 [95% CI, 1.25-2.60]; material and psychological: OR, 1.88 [95% CI, 1.41-2.49]; all 3 domains: OR, 2.25 [95% CI, 1.82-2.78]). Compared with survivors with no hardship, the odds of having an unhealthy lifestyle score were higher among those with material hardship (OR, 1.52 [95% CI, 1.11-2.07]) or psychological hardship (OR, 1.96 [95% CI, 1.31-2.93]), with higher odds among those with hardship in 2 domains (material and behavioral: OR, 1.60 [95% CI, 1.00-2.57]; material and psychological: OR, 2.08 [95% CI, 1.42-3.06]; behavioral and psychological: OR, 2.45 [95% CI, 1.45-4.13]) or all 3 domains (OR, 3.67 [95% CI, 2.69-5.01]). Compared with survivors with no hardship, only those reporting hardship in all 3 domains a had higher odds of a moderately healthy lifestyle score (OR, 1.79 [95% CI, 1.30-2.46]).

### Associations Between Medical Financial Hardship and Nonadherence to Surveillance and Screening Recommendations

**Figure 3.**
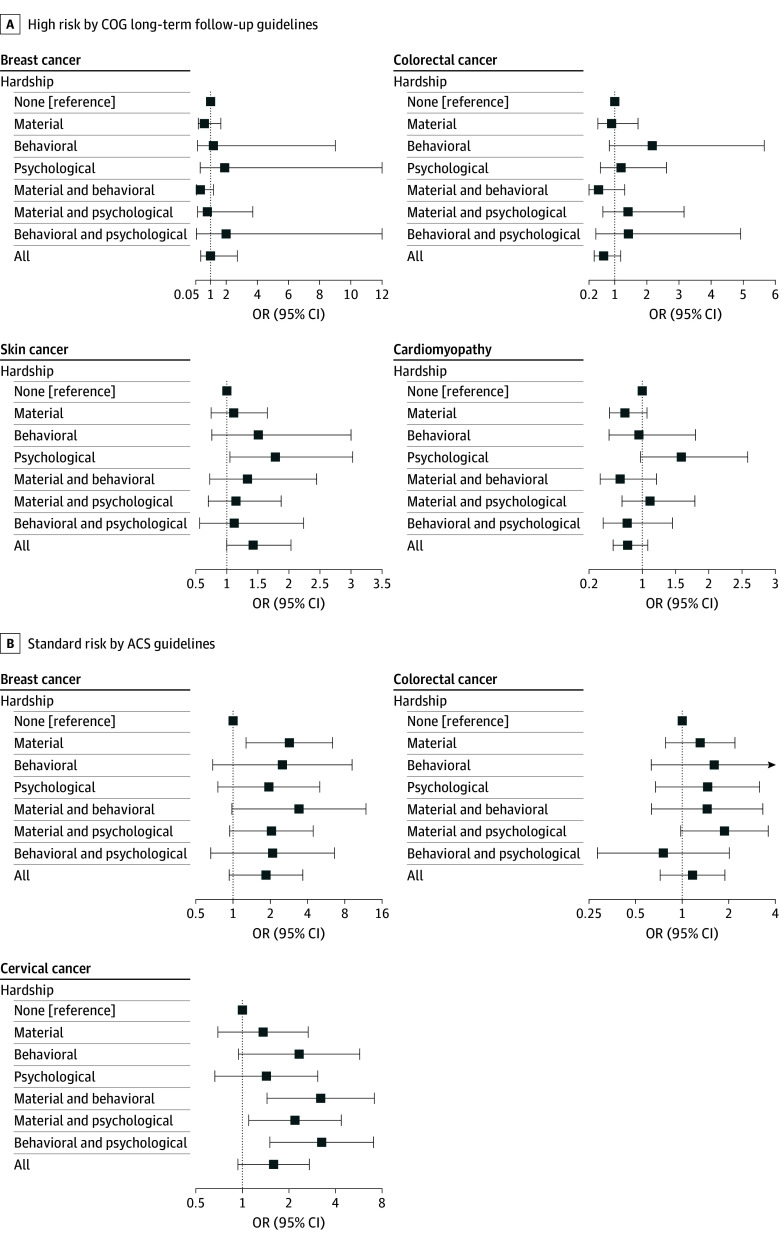
Forest Plots of the Association Between Financial Hardship and Nonadherence to Suggested Surveillance and Screening Recommendations in Childhood Cancer Survivors Considered at High or Standard Risk of Developing Subsequent Malignant Neoplasms or Cardiomyopathy Odds ratios (ORs) and 95% CIs are provided in eTable 6 in Supplement 1. ACS indicates American Cancer Society; COG, Children’s Oncology Group.

 More than half of the survivors (1428 of 2745 [52.0%]) in our cohort were considered to be at a high risk for subsequent skin cancer. Nearly 20% had treatment exposures that increased their risks for breast cancer (265 of 1380 survivors [19.2%]) or colorectal cancer (534 of 2741 survivors [19.4%]) (eTable 4 in Supplement 1). Less than half of the survivors at a high risk of a subsequent malignant neoplasm or cardiomyopathy received appropriate surveillance within the recommended time frame (breast cancer testing, 34 [12.8%]; colorectal cancer testing, 225 [42.1%]; skin cancer testing, 388 [27.1%]; cardiomyopathy testing, 556 [41.5%]) (eTable 5 in Supplement 1). Among survivors at high risk, those reporting psychological hardship were more likely to be nonadherent to subsequent skin cancer surveillance (OR, 1.78 [95% CI, 1.05-3.02]). Among survivors at an average risk for a subsequent malignant neoplasm, having material hardship was associated with an elevated risk of nonadherence to breast cancer screening (OR, 2.85 [95% CI, 1.27-6.38]). With regard to cervical cancer screening, survivors reporting material and behavioral hardship (OR, 3.20 [95% CI, 1.44-7.14]), material and psychological hardship (OR, 2.18 [95% CI, 1.10-4.35]), or behavioral and psychological hardship (OR, 3.25 [95% CI, 1.50-7.04]) were also more likely to be nonadherent (Figure 3; eTable 6 in Supplement 1).

In addition, we separately studied the association between nonadherence to subsequent malignant neoplasm and only material and psychological hardship (eTable 7 in Supplement 1). Among survivors at high risk, no significant associations were noted. Among survivors at an average risk for subsequent malignant neoplasm, having material hardship alone and both material and psychological hardship were associated with an elevated risk of nonadherence to breast cancer screening (OR, 2.79 [95% CI, 1.37-5.67] and 1.80 [95% CI, 1.01-3.19], respectively). With regard to cervical cancer screening, survivors reporting psychological hardship alone and both material and psychological hardship were also more likely to be nonadherent (OR, 1.85 [95% CI, 1.04-3.30] and 1.58 [95% CI, 1.00-2.51], respectively).

## Discussion

This large cohort study of long-term adult survivors of childhood cancer at more than 3 decades since diagnosis found that more than one-half reported medical financial hardship in at least 1 domain. Hardship was associated with unhealthy lifestyle behaviors and nonadherence to specific surveillance recommendations for subsequent malignant neoplasms. Medical financial hardship is a well-documented complication of cancer-directed therapy and can have adverse consequences for survivors’ health status, quality of life,^14,16^ and mortality risk.^32,33^ Thus, considering the similar risks associated with unhealthy lifestyle and nonadherence to surveillance recommendations,^29,34^ our findings suggest a plausible mechanism by which medical financial hardship may contribute to adverse health outcomes among long-term childhood cancer survivors.

The prevalence of unhealthy lifestyle reported in our analysis is consistent with previously published literature.^29,35^ Our analysis adds to prior literature by revealing associations between medical financial hardship and unhealthy lifestyle, which has not been reported in this patient population. While the specific reasons for these associations remain unclear, physical inactivity and abnormal BMI might be associated with financial sacrifices and subsequent inability to afford a healthy lifestyle. Similarly, the associations between behavioral and psychological hardship and unhealthy behaviors may be due to the foregone follow-up care and worry or stress related to not having enough money for essentials. These associations are especially concerning and represent opportunities for interventions because an unhealthy lifestyle is potentially modifiable and associated with late morbidity and mortality among childhood cancer survivors.^29,34^

Adherence rates to surveillance for subsequent malignant neoplasms and cardiomyopathy observed in our study were also consistent with prior literature focused on childhood cancer survivors.^18^ However, to our knowledge, no prior studies have described associations between medical financial hardship and adherence to recommended surveillance in this population, specifically for those at a high risk of developing a subsequent malignant neoplasm or cardiomyopathy due to their prior exposures to radiation or anthracyclines. While no significant associations were observed in our analysis except for lower skin cancer surveillance among those reporting psychological hardship, the lack of clear associations may be due to a highly educated population and having a high proportion of privately insured survivors in our cohort. Nonetheless, our study underscores the need for future work to identify risk factors of low adherence to surveillance recommendations among childhood cancer survivors.

Our findings call for the development of targeted intervention strategies to address both medical financial hardship and lower adherence to a healthy lifestyle and surveillance in long-term childhood cancer survivors. At a minimum, routine screening for medical financial hardship is indicated to potentially identify survivors at risk for adverse outcomes or suboptimal adherence. Additional practice-level changes connecting survivors with needed services and policy-level changes supporting workplace accommodations and optimal insurance coverage are needed to prevent and mitigate medical financial hardship and potential adverse consequences in this at-risk population. Studies implementing interventions for financial navigation^36^ and improving health insurance literacy^37,38^ have also shown benefits in alleviating medical financial hardship. Our findings underscore the importance of providing free or low-cost lifestyle modification programs for survivors facing medical financial hardship, such as LIVESTRONG at the YMCA, which delivers evidence-based exercise and wellness training at no cost, helping to reduce barriers to healthy behaviors and potentially mitigating the compounded risks of unhealthy lifestyles and adverse long-term health outcomes. Similarly, other efforts such as free or subsidized cancer screening through the Centers for Disease Control and Prevention’s National Breast and Cervical Cancer Early Detection Program, insurance navigation support offered by organizations such as CancerCare, or hospital-based financial counseling programs that help survivors access recommended surveillance despite cost barriers may help improve adherence to surveillance among survivors with medical financial hardship.

### Limitations

This study had several limitations. First, we relied on participant self-reported data to define outcomes; therefore, the possibility of participation, healthy survivor, recall, misclassification (of hardship and outcomes), and social desirability biases are of potential concern. Caution needs to be exercised in interpreting the results. However, of note, only a small proportion of survivors who responded to the medical hardship survey did not respond to the follow-up survey assessing lifestyle behaviors and adherence to recommended surveillance, which alleviated the risk of attrition and resultant selection bias to some extent. Second, our study cohort was highly educated, which may have blunted the manifestations of medical financial hardship in this cohort. Third, we did not compare lifestyle and adherence to recommended surveillance between cancer survivors and the general US population. However, previous studies have reported lower adherence to healthy lifestyle and recommended surveillance among childhood cancer survivors compared with the general population.^39^ We used the composite lifestyle score as previously published.^29^ The scoring assigned equal weight to physical activity, smoking, alcohol use, and BMI, which could be a limitation. Of note, the medical hardship survey (2017-2019) and the survey assessing lifestyle behaviors and adherence to recommended surveillance (2020-2022) were performed at different time points, which may have influenced the study findings. Specifically, respondents’ medical financial hardship status, lifestyle, and adherence to guideline-concordant care may have changed over time. However, we were unable to account for these changes due to lack of information on time of onset and resolution of any of these risk factors and outcomes of interest. Therefore, a possible bidirectional association between medical financial hardship and unhealthy lifestyle and nonadherence to cardiomyopathy and subsequent malignant neoplasm surveillance and screening cannot be ruled out. Additionally, our study results may have been influenced by external factors, such as the COVID-19 pandemic given the timing of the survey assessing lifestyle behaviors and adherence to recommended surveillance. Some of the questions in the behavioral hardship domain, such as foregoing medical care, annual visits to a primary care physician, care from a specialist, and survivor care or screening, may have correlated with nonadherence to guideline-concordant care. However, no major differences in findings were noted in the associations between only material and psychological hardship domains and nonadherence to surveillance and screening for subsequent malignant neoplasms and cardiomyopathy (eTable 7 in Supplement 1). Finally, while we studied several social drivers of health, such as education, employment status, annual household income, and living arrangements, we did not study survivors’ location of living and type of survivorship care received at the time of the survey, which may have been associated their medical financial hardship status and adherence to healthy lifestyle and guideline-concordant care.

## Conclusions

This cohort study of adult survivors of childhood cancer found significant associations between medical financial hardship intensity and unhealthy lifestyle behaviors. Specifically, survivors who reported material, behavioral, and psychological hardship were more likely to be physically inactive, to be current smokers, to have an abnormal BMI, and to have composite unhealthy lifestyle scores. The findings underscore the need for identification and implementation of strategies to address specific aspects of medical financial hardship as one approach to improving adherence to recommended lifestyle and surveillance in this population.

## Notes

Supplement 1. **eTable 1.** Survey Domains and Outcomes **eTable 2.** Children’s Oncology Group–Recommended Cancer Surveillance Protocol for High-Risk Populations, Recommended Frequency of Echocardiogram or Comparable Cardiac Imaging, Conversion of Anthracycline Exposures to Doxorubicin, and American Cancer Society–Recommended Cancer Screening Protocol for Standard-Risk Populations **eTable 3.** Association Between Medical Financial Hardship and Lifestyle Behaviors in Childhood Cancer Survivors **eTable 4.** Risk Group of Childhood Cancer Survivors **eTable 5.** Adherence to Suggested Surveillance Recommendations in Childhood Cancer Survivors **eTable 6.** Association Between Financial Hardship Domains (Material, Behavioral, Psychological Alone, and Combined) and Nonadherence to Suggested Surveillance and Screening Recommendations in Childhood Cancer Survivors Who Are Either Considered at a High Risk for Developing Subsequent Neoplasms or Cardiomyopathy **eTable 7.** Association Between Financial Hardship Domains (Material and Psychological Alone and Combined) and Nonadherence to Suggested Surveillance and Screening Recommendations in Childhood Cancer Survivors Who Are Either Considered at a High Risk for Developing Subsequent Neoplasms or Cardiomyopathy **eFigure.** CONSORT Diagram

Supplement 2.
Data Sharing Statement
