# Supplementary material for: Financial Hardship and Nonadherence to Lifestyle and Surveillance in Childhood Cancer Survivors
Source: JAMA Netw Open. 2026 May 29;9(5):e2615527. doi: 10.1001/jamanetworkopen.2026.15527 (PMC13221689; doi:10.1001/jamanetworkopen.2026.15527)
Supplement: Supplement 1. — eTable 1. Survey Domains and Outcomes eTable 2. Children’s Oncology Group–Recommended Cancer Surveillance Protocol for High-Risk Populations, Recommended Frequency of Echocardiogram or Comparable Cardiac Imaging, Conversion of Anthracycline Exposures to Doxorubicin, and American Cancer Society–Recommended Cancer Screening Protocol for Standard-Risk Populations eTable 3. Association Between Medical Financial Hardship and Lifestyle Behaviors in Childhood Cancer Survivors eTable 4. Risk Group of Childhood Cancer Survivors eTable 5. Adherence to Suggested Surveillance Recommendations in Childhood Cancer Survivors eTable 6. Association Between Financial Hardship Domains (Material, Behavioral, Psychological Alone, and Combined) and Nonadherence to Suggested Surveillance and Screening Recommendations in Childhood Cancer Survivors Who Are Either Considered at a High Risk for Developing Subsequent Neoplasms or Cardiomyopathy eTable 7. Association Between Financial Hardship Domains (Material and Psychological Alone and Combined) and Nonadherence to Suggested Surveillance and Screening Recommendations in Childhood Cancer Survivors Who Are Either Considered at a High Risk for Developing Subsequent Neoplasms or Cardiomyopathy eFigure. CONSORT Diagram [file jamanetwopen-e2615527-s001.pdf]

Supplemental Online Content

Bhatt NS, Wang F, Izumi S, et al. Financial hardship and nonadherence to lifestyle and surveillance in childhood cancer survivors. *JAMA Netw Open*. 2026;9(5):e2615527. doi:10.1001/jamanetworkopen.2026.15527

eTable 1. Survey Domains and Outcomes

eTable 2. Children's Oncology Group–Recommended Cancer Surveillance Protocol for High-Risk Populations, Recommended Frequency of Echocardiogram or Comparable Cardiac Imaging, Conversion of Anthracycline Exposures to Doxorubicin, and American Cancer Society–Recommended Cancer Screening Protocol for Standard-Risk Populations

eTable 3. Association Between Medical Financial Hardship and Lifestyle Behaviors in Childhood Cancer Survivors

eTable 4. Risk Group of Childhood Cancer Survivors

eTable 5. Adherence to Suggested Surveillance Recommendations in Childhood Cancer Survivors

eTable 6. Association Between Financial Hardship Domains (Material, Behavioral, Psychological Alone, and Combined) and Nonadherence to Suggested Surveillance and Screening Recommendations in Childhood Cancer Survivors Who Are Either Considered at a High Risk for Developing Subsequent Neoplasms or Cardiomyopathy

eTable 7. Association Between Financial Hardship Domains (Material and Psychological Alone and Combined) and Nonadherence to Suggested Surveillance and Screening Recommendations in Childhood Cancer Survivors Who Are Either Considered at a High Risk for Developing Subsequent Neoplasms or Cardiomyopathy

eFigure. CONSORT Diagram

This supplemental material has been provided by the authors to give readers additional information about their work.

© 2026 Bhatt NS et al. *JAMA Network Open*.

eTable 1. Survey Domains and Outcomes			
**Variable**	**Survey**		**Other**
	**Medical financial hardship (2017-2019)**	**Lifestyle behaviors and adherence to recommended surveillance (2020-2022)**	
**Risk-Factors**			
Material hardship/Financial sacrifices<br>• Reduced spending on vacation or leisure<br>• Reduced spending on large purchases<br>• Delayed or reduced spending on home improvement<br>• Used savings set aside for other purposes<br>• Reduced spending on basics<br>• Paying off medical bills over time<br>• Made a change to living situation<br>• Problems paying medical bills	X		
Behavioral hardship<br>• Forgone any needed medical care<br>• Forgone yearly visit to your primary care doctor<br>• Forgone specialist<br>• Forgone follow-up care<br>• Forgone prescription medicine<br>• Forgone eyeglasses<br>• Forgone mental health counseling<br>• Forgone dental care	X		
Psychological hardship<br>• Worry or stress about having enough money to pay rent or mortgage<br>• Worry or stress about having enough money to buy nutritious meals<br>• Worry or stress about having enough money to pay household utilities, such as water, gas, and electricity	X		
Age at survey	X	X	

© 2026 Bhatt NS et al. *JAMA Network Open*.

Age at diagnosis			Medical records
Sex			CCSS baseline survey
Race/ ethnicity			CCSS baseline survey
Primary cancer diagnosis			Medical records
Treatment			Medical records
Anthracycline exposure and doxorubicin equivalent dose			Medical records
Any radiation exposure (location, dose)			Medical records
Highest education level		X	
Current employment status		X	
Annual household income		X	
Health insurance status		X	
Marital status		X	
Current living arrangement		X	
Chronic health conditions as per CTCAE v4.03		X	
Subsequent neoplasm			Medical records
**Outcomes**:			
Moderate or vigorous intensity exercise		X	
Problematic drinking behavior		X	
Smoking behavior		X	
Body mass index		X	
Late cardiovascular sequelae surveillance		X	
Breast cancer surveillance		X	
Colorectal cancer surveillance		X	
Skin cancer surveillance		X	
Cervical cancer screening		X	

© 2026 Bhatt NS et al. *JAMA Network Open*.

eTable 2. Children's Oncology Group–Recommended Cancer Surveillance Protocol for High-Risk Populations, Recommended Frequency of Echocardiogram or Comparable Cardiac Imaging, Conversion of Anthracycline Exposures to Doxorubicin, and American Cancer Society–Recommended Cancer Screening Protocol for Standard-Risk Populations

**A.** Children's Oncology Group Recommended Cancer Surveillance Protocol for High-Risk Populations		
Organ:	Population at Risk:	Suggested Surveillance:
Breast	Females who received chest, axilla, or total body radiation with potential impact on the breast	Yearly mammogram and adjunct breast MRI beginning 8 years after radiation or at age 25 (whichever occurs later)
Colorectal	Patients who received abdomen, pelvis, spine (lumbar, sacral, whole), or total body radiation with potential to impact the colon/rectum	Colonoscopy every 5 years or multitarget stool DNA test every 3 years beginning 5 years after radiation or at age 30 (whichever occurs later)<br><br>Note: For convenience, if the survivor reported having a surveillance test within last five years, they were considered adherent
Skin	Patients who received any radiation	Yearly skin examination

**B.** Recommended Frequency of Echocardiogram or Comparable Cardiac Imaging		
Anthracycline dose [a]	Radiation dose	Recommended frequency
None	<15 Gy or none	No surveillance
	≥15- <35 Gy	Every 5 years
	≥35 Gy	Every 2 years
<250 mg/m^2	<15 Gy or none	Every 5 years
	≥15 Gy	Every 2 years
≥250 mg/m^2	Any or none	Every 2 years

[a] Cumulative anthracycline dose derived using Doxorubicin equivalent dose calculations

**C.** Conversion of Anthracycline Exposures to Doxorubicin Isotoxic Equivalents as per Feijen EA et al. J Clin Oncol 33:3774-80, 2015	
Anthracycline	Doxorubicin Isotoxic Equivalents
Doxorubicin	1
Daunorubicin	0.5
Epirubicin	0.67
Idarubicin	5
Mitoxantrone	4

© 2026 Bhatt NS et al. *JAMA Network Open*.

**D.** American Cancer Society Recommended Cancer Screening Protocol for Standard Risk Populations	
Breast	Annual mammogram starting at age 45 (can start at 40 if they wish to do so) until age 54 and then every 2 years and continuing as long as the woman is expected to live 10 more years or longer
Cervical [a, b]	Cervical pap smears starting at age 21 and then repeated every 3 years from age 21-29, and every 5 years with a Human Papilloma Virus test from age 30-65 with the potential to stop testing at age 65 if the patient meets specific criteria
Colorectal [a]	Starting at age 45, colonoscopy every 10 years, computerized tomography colonography every 5 years, or flexible sigmoidoscopy every 5 years up to age 75; for patients between 76-85 years of age, the screening should be based on a person's preference, life expectancy, overall health, and prior screening history

[a] For convenience, screening frequency of 5 year for any of the screening test was considered adherent
[b] Survey did not include details regarding an HPV test

© 2026 Bhatt NS et al. *JAMA Network Open*.

eTable 3. Association Between Medical Financial Hardship and Lifestyle Behaviors in Childhood Cancer Survivors [a]

Variable	Physical inactivity (Not meeting physical activity guidelines)		Problematic drinking behavior		Current smoker		Unhealthy BMI		Composite Lifestyle Score			
									Moderately healthy lifestyle		Unhealthy lifestyle	
	**OR**	**(95% CI)**	**OR**	**(95% CI)**	**OR**	**(95% CI)**	**OR**	**(95% CI)**	**OR**	**(95% CI)**	**OR**	**(95% CI)**
**Single Hardship**												
Material hardship only	**1.67**	**(1.29, 2.18)**	0.84	(0.65, 1.09)	0.79	(0.42, 1.49)	**1.47**	**(1.15, 1.88)**	1.19	(0.88, 1.62)	**1.52**	**(1.11, 2.07)**
Behavioral hardship only	1.08	(0.72, 1.63)	0.92	(0.61, 141)	**2.29**	**(1.13, 4.62)**	1.03	(0.67, 1.58)	1.20	(0.72, 2.01)	1.13	(0.66, 1.93)
Psychological hardship only	1.08	(0.80, 1.47)	1.22	(0.90, 1.66)	**3.95**	**(2.42, 6.44)**	1.16	(0.85, 1.58)	1.43	(0.96, 2.14)	**1.96**	**(1.31, 2.93)**
**Combination of Hardship**												
Material and behavioral hardship	0.86	(0.59, 1.25)	1.13	(0.76, 1.67)	**2.23**	**(1.13, 4.40)**	**1.81**	**(1.25, 2.60)**	1.07	(0.66, 1.74)	**1.60**	**(1.00, 2.57)**
Material and psychological hardship	1.26	(0.93, 1.70)	0.99	(0.73, 1.35)	**3.02**	**(1.82, 5.02)**	**1.88**	**(1.41, 2.49)**	1.39	(0.94, 2.05)	**2.08**	**(1.42, 3.06)**
Behavioral and psychological hardship	1.09	(0.74, 1.60)	1.08	(0.73, 1.59)	**4.19**	**(2.39, 7.36)**	1.10	(0.75, 1.62)	1.46	(0.85, 2.50)	**2.45**	**(1.45, 4.13)**
All three types of hardship	**1.31**	**(1.04, 1.65)**	1.05	(0.83, 1.31)	**3.70**	**(2.50, 5.46)**	**2.25**	**(1.82, 2.78)**	**1.79**	**(1.30, 2.46)**	**3.67**	**(2.69, 5.01)**

OR: odds ratio, CI: confidence interval

[a] Each model adjusted for age at the time of most recent survey, sex, race and ethnicity, highest education level, and chronic health burden

© 2026 Bhatt NS et al. *JAMA Network Open*.

eTable 4. Risk Group of Childhood Cancer Survivors

Characteristic	N (%)
**Breast cancer risk (n=1380)**	
COG high risk	265 (19.2%)
ACS standard risk	337 (24.4%)
Not at risk	720 (52.1%)
Treatment Info missing	58 (4.2%)
**Colorectal cancer risk (n=2741)**	
COG high risk	534 (19.4%)
ACS standard risk	743 (27.1%)
Not at risk	1369 (49.9%)
Treatment Info missing	95 (3.4%)
**Skin cancer risk (n=2745)**	
COG high risk	1428 (52.0%)
Not at risk	1183 (43.1%)
Treatment Info missing	134 (4.8%)
**Cervical cancer risk (n=1453)**	
ACS standard risk	1185 (81.5%)
Not at risk*	268 (18.4%)
**Cardiomyopathy risk group (n=2693)**	
None	1083 (40.2%)
2 years	658 (24.4%)
5 years	681 (25.2%)
Treatment Info missing	271 (10.0%)

COG: Children's Oncology Group; ACS: American Cancer Society
*Included survivors with a history of a hysterectomy

© 2026 Bhatt NS et al. *JAMA Network Open*.

eTable 5. Adherence to Suggested Surveillance Recommendations in Childhood Cancer Survivors

Characteristic	High risk		Standard risk	
	N	(%)	N	(%)
**Breast cancer testing**				
Had Mammogram and MRI test within recommended period	34	(12.8%)		
Had Mammogram or MRI test within recommended period	80	(30.1%)	201	(59.6%)
Had Mammogram or MRI test, but not within recommended period	89	(33.5%)	105	(31.1%)
Never had test	50	(18.8%)	22	(6.5%)
Don't know	12	(4.5%)	9	(2.6%)
**Colorectal cancer testing**				
Had test within recommended period	225	(42.1%)	366	(49.2%)
Had test, but not within recommended period	82	(15.3%)	116	(15.6%)
Never had test	181	(33.9%)	221	(29.7%)
Don't know	46	(8.6%)	40	(5.3%)
**Skin cancer testing**				
Had test within recommended period	388	(27.1%)		
Had test, but not within recommended period	418	(29.2%)		
Never had test	480	(33.6%)		
Don't know	142	(9.9%)		
**Cervical cancer testing**				
Had test within recommended period			1007	(84.9%)
Had test, but not within recommended period			91	(7.6%)
Never had test			46	(3.8%)
Don't know			41	(3.4%)
**Cardiomyopathy testing**				
Had test within recommended period	556	(41.5%)		
Had test, but not within recommended period	496	(37.0%)		
Never had test	183	(13.6%)		
Don't know	104	(7.7%)		

© 2026 Bhatt NS et al. *JAMA Network Open*.

eTable 6. Association Between Financial Hardship Domains (Material, Behavioral, Psychological Alone, and Combined) and Nonadherence to Suggested Surveillance and Screening Recommendations in Childhood Cancer Survivors Who Are Either Considered at a High Risk for Developing Subsequent Neoplasms or Cardiomyopathy

A: Association between financial hardship and nonadherence to subsequent neoplasms and cardiomyopathy surveillance in survivors considered high-risk for developing these outcomes as per the Children's Oncology Group Long-Term Follow-up Guidelines [a]

Risk Group	COG High Risk Survivors							
Variable	Breast cancer		Colorectal cancer		Skin cancer		Cardiomyopathy	
	**OR**	**(95% CI)**	**OR**	**(95% CI)**	**OR**	**(95% CI)**	**OR**	**(95% CI)**
**Single Hardship**								
Material hardship only	0.59	(0.21, 1.66)	0.90	(0.47, 1.72)	1.11	(0.74, 1.65)	0.73	(0.50, 1.07)
Behavioral hardship only	1.18	(0.15, 9.00)	2.16	(0.83, 5.65)	1.50	(0.75, 3.00)	0.94	(0.50, 1.79)
Psychological hardship only	1.90	(0.33, 10.83)	1.19	(0.54, 2.61)	**1.78**	**(1.05, 3.02)**	1.58	(0.97, 2.58)
**Combination of Hardship**								
Material and behavioral hardship	0.34	(0.10, 1.17)	0.49	(0.18, 1.30)	1.33	(0.72, 2.44)	0.66	(0.36, 1.21)
Material and psychological hardship	0.78	(0.16, 3.70)	1.41	(0.63, 3.15)	1.14	(0.70, 1.87)	1.11	(0.69, 1.79)
Behavioral and psychological hardship	1.99	(0.09, 43.72)	1.42	(0.41, 4.92)	1.12	(0.56, 2.23)	0.77	(0.41, 1.45)
All three types of hardship	0.99	(0.36, 2.72)	0.65	(0.35, 1.18)	1.42	(0.99, 2.03)	0.78	(0.56, 1.08)

B: Association between financial hardship and nonadherence to subsequent neoplasms screening in survivors considered standard risk for developing these outcomes as per the American Cancer Society Guidelines [a]

Risk Group	ACS Standard Risk Survivors					
Variable	Breast cancer		Colorectal cancer		Cervical cancer	
	**OR**	**(95% CI)**	**OR**	**(95% CI)**	**OR**	**(95% CI)**
**Single Hardship**						
Material hardship only	**2.85**	**(1.27, 6.38)**	1.30	(0.78, 2.19)	1.36	(0.69, 2.66)
Behavioral hardship only	2.50	(0.68, 9.16)	1.61	(0.63, 4.11)	2.32	(0.94, 5.74)
Psychological hardship only	1.94	(0.75, 5.02)	1.46	(0.67, 3.16)	1.43	(0.66, 3.07)
**Combination of Hardship**						
Material and behavioral hardship	3.41	(0.98, 11.88)	1.45	(0.63, 3.32)	**3.20**	**(1.44, 7.14)**
Material and psychological hardship	2.04	(0.93, 4.44)	1.87	(0.97, 3.59)	**2.18**	**(1.10, 4.35)**
Behavioral and psychological hardship	2.08	(0.66, 6.59)	0.75	(0.28, 2.01)	**3.25**	**(1.50, 7.04)**
All three types of hardship	1.84	(0.93, 3.66)	1.16	(0.72, 1.88)	1.59	(0.93, 2.70)

OR: odds ratio, CI: confidence interval

[a] Each model adjusted for age at the time of most recent survey, sex, race and ethnicity, highest education level, and chronic health burden

© 2026 Bhatt NS et al. *JAMA Network Open*.

eTable 7. Association Between Financial Hardship Domains (Material and Psychological Alone and Combined) and Nonadherence to Suggested Surveillance and Screening Recommendations in Childhood Cancer Survivors Who Are Either Considered at a High Risk for Developing Subsequent Neoplasms or Cardiomyopathy

A: Association between financial hardship and nonadherence to subsequent neoplasms and cardiomyopathy surveillance in survivors considered high-risk for developing these outcomes as per the Children's Oncology Group Long-Term Follow-up Guidelines [a]

**Risk Group**	**COG High Risk Survivors**							
**Variable**	**Breast cancer**		**Colorectal cancer**		**Skin cancer**		**Cardiomyopathy**	
	**OR**	**(95% CI)**	**OR**	**(95% CI)**	**OR**	**(95% CI)**	**OR**	**(95% CI)**
**Single Hardship**								
Material hardship only	0.48	(0.20, 1.14)	0.69	(0.40, 1.21)	1.13	(0.79, 1.60)	0.72	(0.52, 1.00)
Psychological hardship only	2.33	(0.43, 12.54)	1.16	(0.59, 2.28)	1.47	(0.95, 2.28)	1.24	(0.83, 1.85)
**Combination of Hardship**								
Material and psychological hardship	0.94	(0.38, 2.35)	0.77	(0.46, 1.28)	1.29	(0.94, 1.76)	0.86	(0.65, 1.16)

B: Association between financial hardship and nonadherence to subsequent neoplasms screening in survivors considered standard risk for developing these outcomes as per the American Cancer Society Guidelines [a]

**Risk Group**	**ACS Standard Risk Survivors**					
**Variable**	**Breast cancer**		**Colorectal cancer**		**Cervical cancer**	
	**OR**	**(95% CI)**	**OR**	**(95% CI)**	**OR**	**(95% CI)**
**Single Hardship**						
Material hardship only	**2.79**	**(1.37, 5.67)**	1.30	(0.82, 2.06)	1.65	(0.95, 2.84)
Psychological hardship only	1.85	(0.85, 4.00)	1.10	(0.59, 2.06)	**1.85**	**(1.04, 3.30)**
**Combination of Hardship**						
Material and psychological hardship	**1.80**	**(1.01, 3.19)**	1.32	(0.87, 1.99)	**1.58**	**(1.00, 2.51)**

OR: odds ratio, CI: confidence interval

[a] Each model adjusted for age at the time of most recent survey, sex, race and ethnicity, highest education level, and chronic health burden

© 2026 Bhatt NS et al. *JAMA Network Open*.

eFigure. CONSORT Diagram

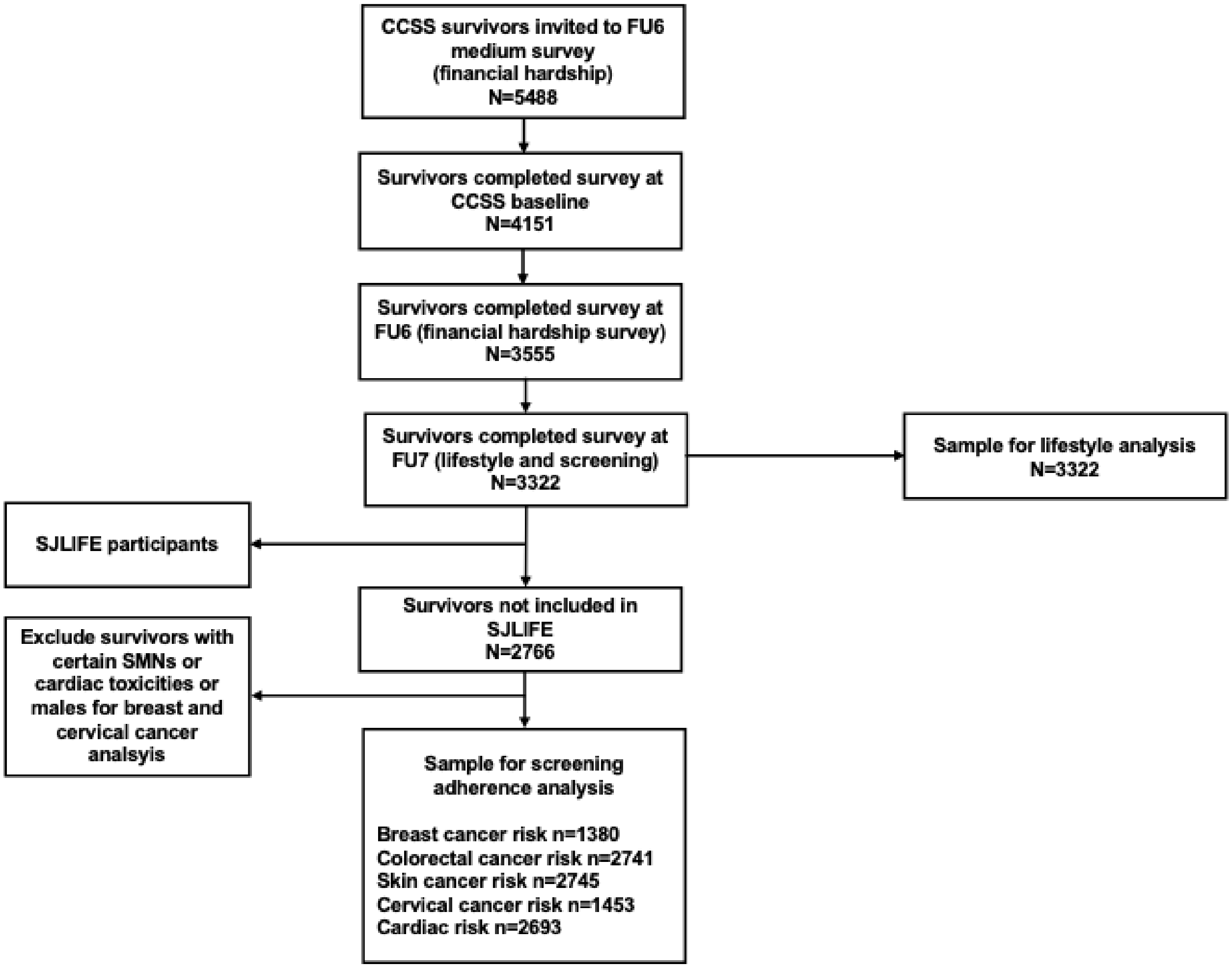

CCSS: Childhood Cancer Survivor Study; FU6: follow-up 6 survey; FU7: follow-up 7 survey; SJLIFE: St. Jude Lifetime Cohort; SMN: subsequent malignant neoplasms

© 2026 Bhatt NS et al. *JAMA Network Open*.
