# Supplementary material for: Financial Hardship and Nonadherence to Lifestyle and Surveillance in Childhood Cancer Survivors
Source: JAMA Netw Open. 2026 May 29;9(5):e2615527. doi: 10.1001/jamanetworkopen.2026.15527 (PMC13221689; doi:10.1001/jamanetworkopen.2026.15527)
Supplement: Supplement 2. — Data Sharing Statement [file jamanetwopen-e2615527-s002.pdf]

Data Sharing Statement

Data
Data available: Yes
Data types: Deidentified participant data
How to access data: CCSS data are publicly available on dbGaP at https://www.ncbi.nlm.nih.gov/gap/ through its accession number phs001327.v2.p1. and on the St Jude Survivorship Portal within the St. Jude Cloud at https://survivorship.stjude.cloud/.
When available: With publication

Supporting Documents
Document types: None

Additional Information
Who can access the data: For this utilization, a research Application Of Intent followed by an Analysis Concept Proposal must be submitted for evaluation by the CCSS Publications Committee.
Types of analyses: Analysis for research purposes approved by the CCSS publication committee.
Mechanisms of data availability: For this utilization, a research Application Of Intent followed by an Analysis Concept Proposal must be submitted for evaluation by the CCSS Publications Committee.
